# Hepatotoxicity Induced by *Sophora flavescens* and Hepatic Accumulation of Kurarinone, a Major Hepatotoxic Constituent of *Sophora flavescens* in Rats

**DOI:** 10.3390/molecules22111809

**Published:** 2017-10-25

**Authors:** Peng Jiang, Xiuwen Zhang, Yutong Huang, Nengneng Cheng, Yueming Ma

**Affiliations:** 1 Department of Pharmacology, School of Pharmacy, Fudan University, Shanghai 201203, China; jiangpeng0640417@126.com (P.J.); 11307120316@fudan.edu.cn (Y.H.); 2 Department of Pharmacy, Eye Ear Nose Throat Hospital of Fudan University, Shanghai 200031, China; 13211030035@fudan.edu.cn; 3 Department of Pharmacology, Shanghai University of Traditional Chinese Medicine, Shanghai 201203, China; mayueming_117@hotmail.com

**Keywords:** *Sophora flavescens*, kurarinone, hepatotoxicity, metabolomics, steatosis, transporter

## Abstract

Our previous study showed that kurarinone was the main hepatotoxic ingredient of *Sophora flavescens*, accumulating in the liver. This study characterized the mechanism of *Sophora flavescens* extract (ESF) hepatotoxicity and hepatic accumulation of kurarinone. ESF impaired hepatic function and caused fat accumulation in the liver after oral administration (1.25 and 2.5 g/kg for 14 days in rats). Serum metabolomics evaluation based on high-resolution mass spectrometry was conducted and real-time PCR was used to determine the expression levels of CPT-1, CPT-2, PPAR-α, and LCAD genes. Effects of kurarinone on triglyceride levels were evaluated in HL-7702 cells. Tissue distribution of kurarinone and kurarinone glucuronides was analyzed in rats receiving ESF (2.5 g/kg). Active uptake of kurarinone and kurarinone glucuronides was studied in OAT2-, OATP1B1-, OATP2B1-, and OATP1B3-transfected HEK293 cells. Our results revealed that after oral administration of ESF in rats, kurarinone glucuronides were actively transported into hepatocytes by OATP1B3 and hydrolyzed into kurarinone, which inhibited fatty acid β-oxidation through the reduction of l-carnitine and the inhibition of PPAR-α pathway, ultimately leading to lipid accumulation and liver injury. These findings contribute to understanding hepatotoxicity of kurarinone after oral administration of ESF.

## 1. Introduction

**Figure 1:**
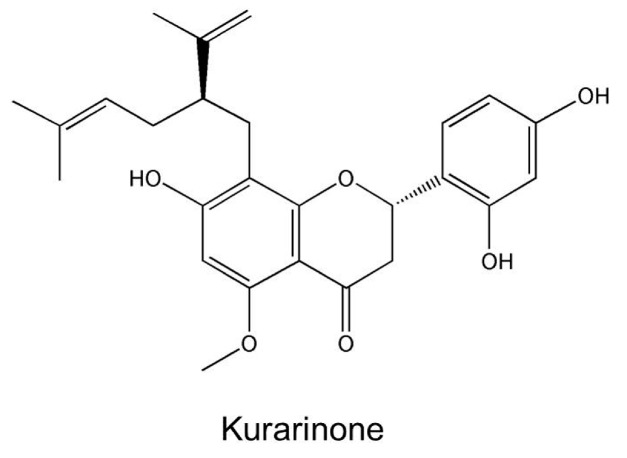
The chemical structure of kurarinone.

Traditional Chinese medicine (TCM) has been used to treat diseases for thousands of years. TCM preparations are widely available and often regarded by the public as harmless remedies for a great variety of illnesses. However, some of these herbal preparations are associated with adverse effects, including hepatotoxicity [1,2,3]. Recently, examples of herb-induced liver injury (HILI) were reported [4,5]. *Sophora flavescens,* Fabaceae, is widely used in TCM for the treatment of viral hepatitis, cancers, viral myocarditis, gastrointestinal hemorrhage, and skin diseases [6]. Many herbal products contain *Sophora flavescens* including Xiaoyin tablets, Shiduqing capsules, Kushen tablets, Danggui Kushen Wan, and Zhixue capsules. However, *Sophora flavescens* was indicated as the hepatotoxic ingredient of Zhixue capsules, which were recalled from the market because of serious hepatotoxic adverse events [7]. In our previous study, kurarinone, a flavonoid aglycone, was identified as the major hepatotoxic constituent of *Sophora flavescens* [8] and hepatic accumulation of kurarinone was detected (Figure 1). However, the mechanisms of extract of *Sophora flavescens* (ESF)-induced hepatotoxicity and kurarinone accumulation remained unclear.

Systematic and comprehensive evaluation of HILI is complicated by high complexity of the ingredients, and HILI mechanisms are not well-characterized [9]. Metabolomics is a global understanding of metabolite profiling in living systems and dynamic responses to the changes of endogenous and exogenous factors and an important tool for research of complex systems [10]. Metabolomics describes metabolic changes in a system caused by interventions in a holistic context. Therefore, metabolomics may assist in understanding the mechanisms of action of TCM preparations, which are complex systems [11].

Intracellular concentrations of drugs and their metabolites are often important determinants of toxicity. Hepatic drug distribution is affected by many factors, including physicochemical properties, uptake/efflux transporters, organelle sequestration, and metabolism [12]. Hepatic transporters play a crucial role in the cellular influx and efflux of endogenous and xenobiotic compounds. Uptake transporters participate in hepatic accumulation of substances, especially OCT1, OAT2, NTCP, OATP1B1, OATP2B1, and OATP1B3 transporters located at the basolateral membrane of hepatocytes. Kurarinone and its metabolites are acidic anionic compounds; however, whether their transport is mediated by organic anion transporters (OATs) and organic anion transporting polypeptides (OATPs) is unclear.

In the present study, metabolomics evaluation was carried out to characterize the mechanism of *Sophora flavescens* hepatotoxicity, whereas transport of kurarinone and its metabolites was analyzed to investigate the possible causes of kurarinone hepatic accumulation.

## 2. Results

### 2.1. ESF Induces Hepatic Lipid Accumulation and Liver Injury

**Figure 2:**
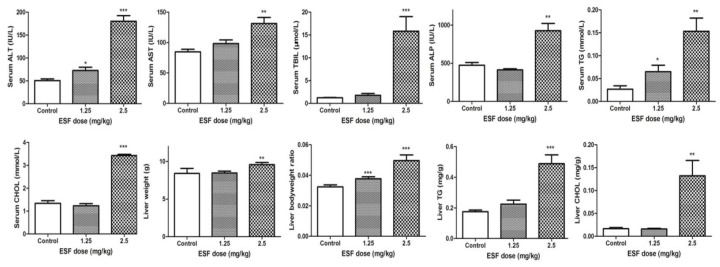
Effect of extract of *Sophora flavescens* on biochemical parameters in male rats for 2 weeks. ALT, Alanine aminotransferase; AST, Aspartate transaminase; TBIL, total bilirubin; ALP, alkaline phosphatase; TG, Triglyceride; CHOL, total cholesterol. * *p* < 0.05, ** *p* < 0.01, *** *p* < 0.001 versus vehicle-treated group.

Hepatic TG and CHOL levels and biochemistry indices in the 2.5 g/kg ESF group were significantly higher, compared to control, indicating ESF-induced liver injury. However, no significant differences were observed between biochemistry indices of the 1.25 g/kg ESF group and control, with the exception of TG and ALT (Figure 2).

**Figure 3:**
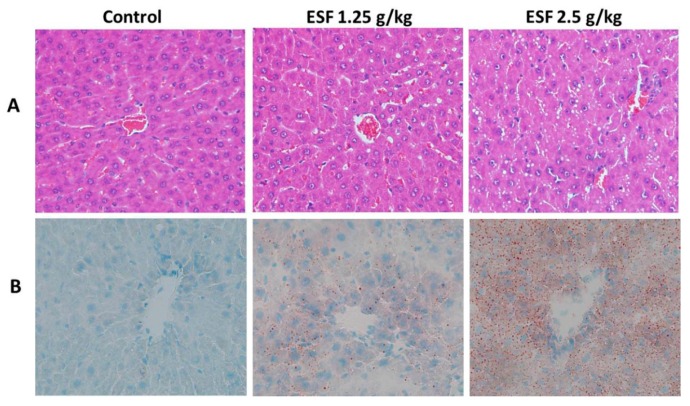
Hepatic histopathology of rats administered with extract of *Sophora flavescens* for 2 weeks (200×). (**A**) Hematoxylin and eosin staining; (**B**) Oil-red O staining.

Photomicrographs of the liver showed remarkable fat accumulation with numerous micro and macro vesicles in the portal area of the 2.5 g/kg ESF-treated rats compared to control. The 1.25 g/kg ESF-treated group exhibited moderate fatty infiltration (Figure 3). Oil-red O staining of the liver revealed significant accentuation of fat staining in the 2.5 g/kg ESF group, whereas only mild hepatic fat accumulation was found in rats treated with 1.25 g/kg ESF. Steatosis was significant and dose-dependent (Figure 3).

### 2.2. Metabolomics Study of the ESF Effects in Rats

#### 2.2.1. Data Acquisition

Representative total ion chromatograms (TICs) of the groups are shown in Supplementary Materials Figures S1 and S2. A visual inspection of the chromatograms revealed differences between the groups. After aligning peaks, 2312 (ESI−) and 3122 (ESI+) spectral features were obtained from each serum sample analyzed by LC/MS. After screening with the “80% rule” and “RSD ≤ 30%” criteria [13], 1352 (ESI−) and 1480 (ESI+) metabolite variables (*m*/*z*—retention time pairs) were obtained for the serum samples.

**Figure 4:**
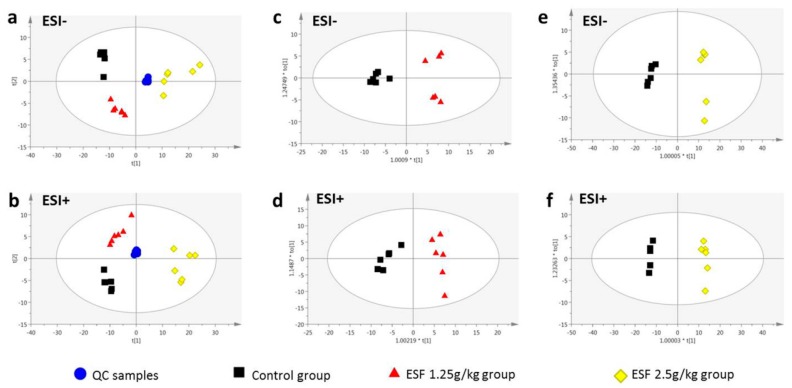
Scores plots of principal components analysis (**a**,**b**) and orthogonal partial least squares discriminant analysis (**c**–**f**) models based on the data of serum samples of rats dosed with extract of *Sophora flavescens* for two weeks from UPLC/MS profiling data with the statistical parameters as follows: (**a**) *R*^2^*X* = 0.969, *Q*^2^ = 0.918; (**b**) *R*^2^*X* = 0.875, *Q*^2^ = 0.775; (**c**) *R*^2^*X* = 0.781, *R*^2^*Y* = 0.962, *Q*^2^ = 0.906; (**d**) *R*^2^*X* = 0.689, *R*^2^*Y* = 0.945, *Q*^2^ = 0.867; (**e**) *R*^2^*X* = 0.908, *R*^2^*Y* = 0.993, *Q*^2^ = 0.978; (**f**) *R*^2^*X* = 0.858, *R*^2^*Y* = 0.996, *Q*^2^ = 0.982. *ESI−* negative electrospray ionization, *ESI+* positive electrospray ionization, *QC* quality control.

In the UPLC/MS positive and negative ion mode, PCA score plots of serum samples from the three groups showed three principal components (*R*^2^*X* = 0.875 and 0.969, respectively). Good separation was obtained between the control and ESF-groups (Figure 4), indicating significant biochemical changes induced by ESF. QC samples clustered tightly in both modes, illustrating the stability of the LC/MS platform throughout the analysis.

Supervised OPLS-DA was applied to separate groups of serum samples and aid the screening of marker metabolites. Figure 4 displays the results of the OPLS-DA, showing an appreciable separation of the data pertaining to the two groups (control vs. 1.25 g/kg ESF group or control vs. 2.5 g/kg ESF group) in positive and negative ion mode. Based on the VIP values (threshold > 1) of OPLS-DA and *p* values (threshold < 0.05) of nonparametric Kruskal-Wallis rank sum test, 196 (ESI+) and 192 (ESI−) metabolite variables (control vs. 1.25 g/kg ESF group) and 187 (ESI+) and 225 (ESI−) differential metabolites (control vs. 2.5 g/kg ESF group) were obtained for further identification.

#### 2.2.2. Identification of Differential Metabolites and Analysis of Metabolic Pathways

**Figure 5:**
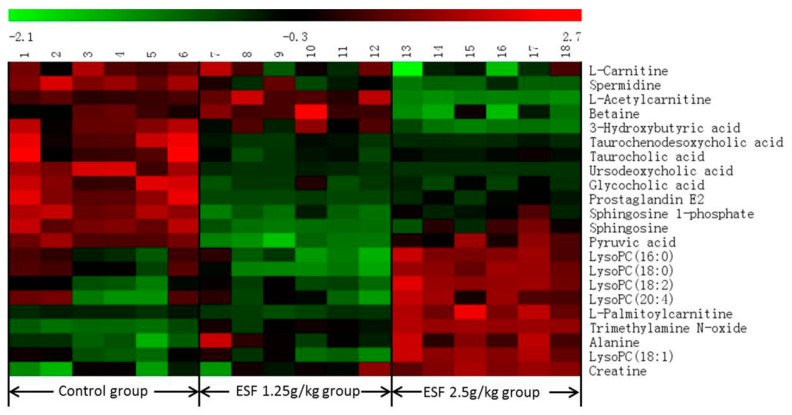
The heat map denoting fold changes of the 22 differential metabolites in different groups of rats dosed with extract of *Sophora flavescens* for two weeks.

Twenty-two differential metabolites were significantly altered and are summarized in Table 1 with corresponding retention times, detected *m*/*z*, VIP, trend, related metabolic pathways, *p*-values, and ESI mode. Seven of the 22 marker metabolites were identified by authentic standards, five were identified by LipidSearch software, and the others were deduced using accurate molecular weights, MS/MS fragments, and metabolomics databases. Figure 5 illustrates the heat map of the changes in differential metabolites in the three groups. As shown in Table 1, metabolic pathways mainly correlated with lipid (fatty acid, glycerophospholipid, and phospholipid), bile acids, and amino acids metabolism.

### 2.3. Quantitative Real-Time PCR

**Figure 6:**
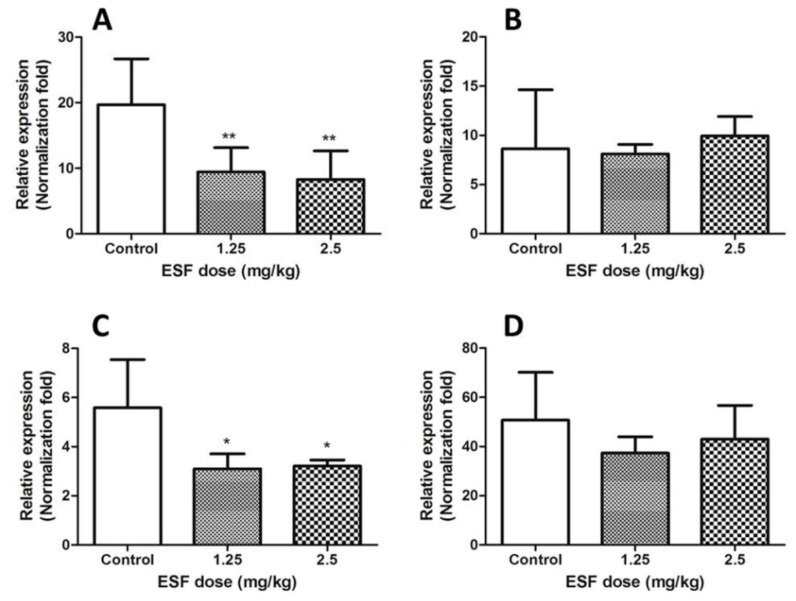
Effect of ESF on liver CPT-1, CPT-2, PPAR-α and LCAD mRNA in rats for two weeks. (**A**) The mRNA level of hepatic CPT-1; (**B**) The mRNA level of hepatic CPT-2; (**C**) The mRNA level of hepatic PPAR-α; (**D**) The mRNA level of hepatic LCAD. Data are expressed as mean ± SD of each group (n = 6 per group). * *p* < 0.05, ** *p* < 0.01, compared with control group.

Steatosis has been linked to inhibition of fatty acid oxidation [14]. By associating identified metabolites with fatty acid metabolism, the metabolic network of ESF-induced fatty acid β-oxidation was constructed. Expression levels of CPT-1, CPT-2, PPAR-α, and LCAD genes are shown in Figure 6.

### 2.4. Effects of Kurarinone on Triglyceride Levels in HL 7702 Cells

**Figure 7:**
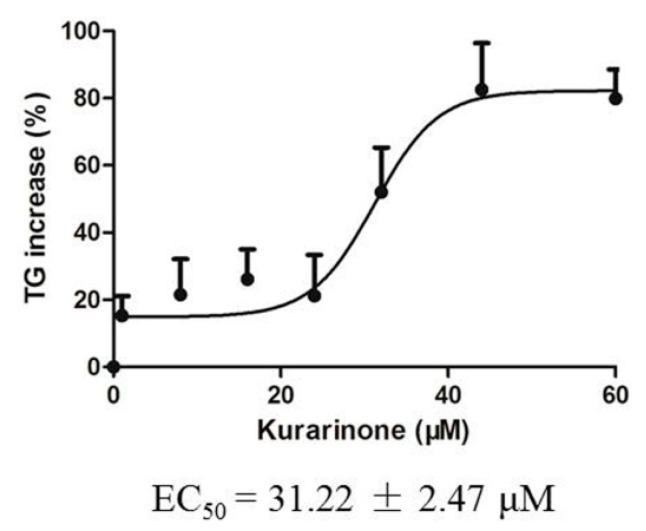
Increase of triglyceride in HL 7702 cells exposed to kurarinone for 24 h.

Triglyceride levels in HL 7702 cells were determined after exposure to kurarinone for 24 h. Triglycerides accumulated dose-dependently. EC_50_ values are shown in Figure 7.

### 2.5. Tissue Distribution of Kurarinone and Kurarinone Glucuronides

**Figure 8:**
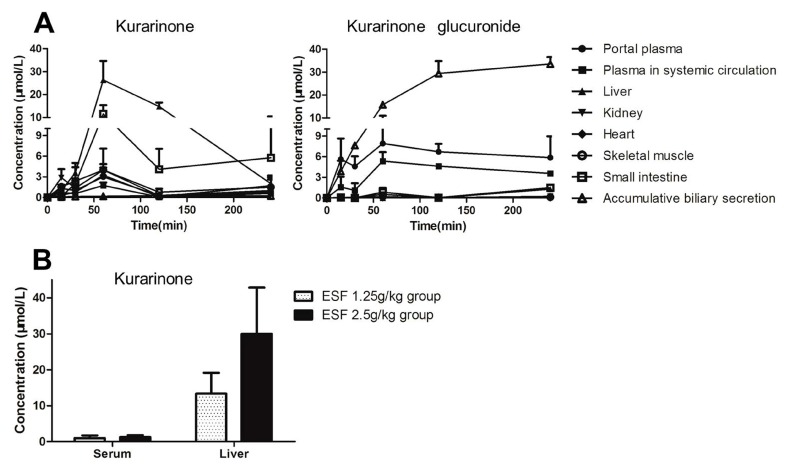
Kurarinone and kurarinone glucuronide levels in fluids and tissues of rats dosed with extract of *Sophora flavescens*(ESF). (**A**) Tissue distribution of kurarinone and kurarinone glucuronide after oral administration of ESF (2.5 g/kg) in rats (mean ± S.D., *n* = 3); (**B**) Kurarinone levels in serum and liver of ESF-treated rats for 2 weeks (mean ± S.D., *n* = 6).

Kurarinone and kurarinone glucuronide levels in fluids and tissues of ESF-treated rats are shown in Figure 8. Kurarinone glucuronide was the dominant form of kurarinone in plasma and bile, whereas kurarinone was predominant in visceral organs. Kurarinone levels in the liver of rats administered with ESF (2.5 g/kg) for two weeks were close to the the concentration of kurarinone in the liver 1 h after dosing. The AUC_0–4h_ values are shown in Table 2. Kurarinone exposure of liver was much higher than that of plasma.

### 2.6. Transport Study of Kurarinone and Kurarinone Glucuronides

**Figure 9:**
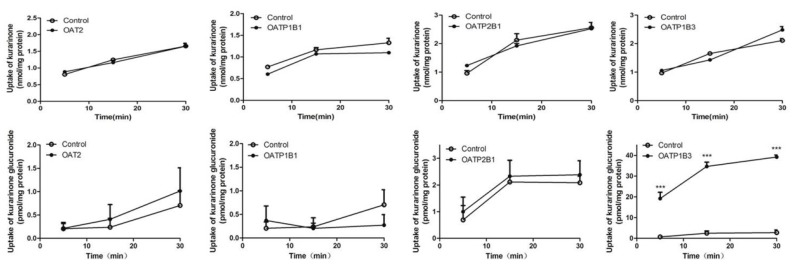
Time courses of uptake of kurarinone and kurarinone glucuronide by OAT2-, OATP1B1-, OATP2B1-, and OATP1B3- transfected HEK293 cells. (mean ± S.D., *n* = 3). *** *p* < 0.001 versus control.

Time-dependent uptakes are shown in Figure 9. No significant differences between the uptake of kurarinone glucuronides by OAT2-, OATP1B1- and OATP2B1-HEK293 cells and that of the vector control cells were observed. However, the uptake of kurarinone glucuronides by OATP1B3-HEK293 cells was significantly higher than that by vector control cells at 37 °C. No significant differences between OAT2, OATP1B1, OATP2B1, and OATP1B3-HEK293 cells and control cells were observed for kurarinone (Figure 9).

## 3. Discussion

Zhixue capsules were reported to induce hepatotoxicity in the clinic [15]. Our previous study showed that *Sophora flavescens* was the hepatotoxic herbal ingredient of Zhixue capsules, inducing numerous vesicles in the portal area of rats receiving 1.25 and 2.5 g/kg ESF twice daily [8]. In this study, liver injury induced in rats administered ESF (1.25 and 2.5 g/kg once a day for 14 days) was characterized by hepatic lipid accumulation.

**Figure 10:**
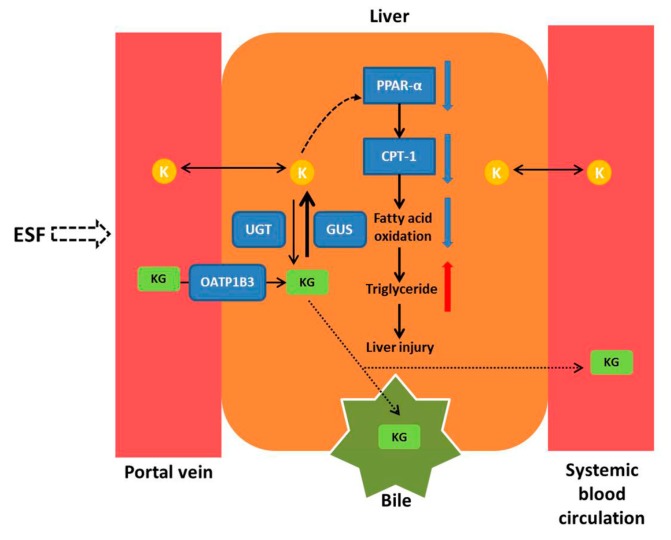
The mechanism of hepatotoxicity induced by *Sophora flavescens*. Key: K, kurarinone; KG, kurarinone glucuronides; UGT, UDP-glucuronosyltransferase; GUS, β-glucuronidase; PPAR-α, peroxisome proliferator-activated receptor-alpha; CPT-1, carnitine palmitoyltransferase-1.

Serum metabolomics results of rats after oral exposure to ESF showed a disturbance of lipid metabolism, especially for differential metabolites associated with fatty acid metabolism. Decreased levels of l-carnitine, l-acetylcarnitine, and 3-hydroxybutyric acid and increased levels of l-palmitoylcarnitine indicated the inhibition of fatty acid oxidation that is the primary cause of steatosis [14]. PPAR-α is a nuclear receptor that controls lipid metabolism by regulating the expression of lipogenic genes, such as CPT-1 [16]. Expression of PPAR-α, which is predominantly expressed in the liver, decreased hepatic lipid accumulation and protected mice from high fat-induced hepatic steatosis [17]. CPT-1 is the rate-limiting enzyme of mitochondria-mediated fatty acids β-oxidation, facilitating mitochondrial uptake of long-chain fatty acids. Real-time PCR and western blot analysis indicated that ESF inhibited the expression of PPAR-α, increased the consumption of l-carnitine, and down-regulated CPT-1 expression, thus reducing fatty acid oxidation and hepatic lipolysis, which ultimately led to hepatic lipid accumulation and liver injury (Figure 10). Additionally, excessive levels of lysophosphatidylcholine (LysoPC), a phosphatidylcholine cleavage product, have been implicated in hepatocellular apoptosis [18]. This is supported by recent data suggesting that LysoPC is a death effector for lipoapoptosis of hepatocytes, which may contribute to steatosis and hepatocyte lipoapoptosis [19].

Kurarinone is the major hepatotoxic compound of *Sophora flavescens* [8]. In this study, kurarinone increased triglyceride levels in HL 7702 cells, with EC_50_ close to the concentration of kurarinone in the liver 1 h after dosing, supporting kurarinone as the hepatotoxic compound of *Sophora flavescens* that leads to triglycerides accumulation in hepatocytes. Other compounds such as sophoraflavanone G [8] may also contribute to the toxicity of ESF. Further research needs to be conducted to investigate the role of other compounds.

In this study, kurarinone glucuronide was the dominant form of kurarinone in the portal vein, indicating that kurarinone was largely metabolized into kurarinone glucuronides after passive transport (log *p* = 5.6) into the intestinal mucosa. Kurarinone glucuronides were transported into the liver by OATP1B3, a specific uptake transporter exclusively located on the basal side of hepatocytes. In the liver, metabolic conjugation by phase II enzymes (e.g., UGTs) and metabolic hydrolysis by β-glucuronidases occurred concurrently. Kurarinone glucuronides were rapidly hydrolyzed to kurarinone, of which the concentration in hepatocytes was much higher than in plasma, reaching levels causing liver injury (Figure 10). In addition, kurarinone glucuronides were the dominant form of kurarinone in bile and systemic blood circulation, suggesting that kurarinone glucuronides might be excreted into bile by efflux transporters such as multi-drug resistance protein 2 (MRP2) and breast cancer resistance protein (BCRP) and into systemic blood circulation by other MRPs, as glucuronides are often substrates of MRPs [20,21] and BCRP [22]. However, further studies are required to uncover whether kurarinone glucuronides are substrates of these efflux transporters. Moreover, modulation of kurarinone metabolism and its involvement in changes seen in vivo may need to be investigated.

The present study suggests genetic polymorphism of OATP1B3 or combination of *Sophora flavescens* with OATP1B3 inducers might affect the occurrence of *Sophora flavescens* hepatotoxicity in the clinic; however, further research is required.

## 4. Materials and Methods

### 4.1. Chemicals and Reagents

Pyruvic acid reference standard was provided by Shanghai Yuanye Bio-Technology Co., (Shanghai, China). Kurarinone was supplied by WuXi PharmaTech (Shanghai, China). Osalmide was purchased from Shanghai JinYi Fine Chemical Co., Ltd. (Shanghai, China). Ursodeoxycholic acid (UDCA), glycocholic acid (GCA), taurocholic acid (TCA), taurochenodeoxycholic acid (TCDCA), uric acid, hippuric acid, creatine, uridine 5′-diphosphoglucuronic acid (UDPGA), β-glucuronidase, d-saccharic acid 1,4-lactone monohydrate (DGA), and alamethicin were purchased from Sigma-Aldrich Co. (St. Louis, MO, USA). The purity of all reference compounds was determined to be >98% by HPLC analysis. *Sophora flavescens* roots were purchased from the Shanghai Tong Ji Tang Pharmaceutical Co., Ltd. (Shanghai, China). HPLC-grade acetonitrile and methanol were purchased from Burdick & Jackson (Muskegon, MI, USA). Formic acid (HPLC-grade) was purchased from CNW Technologies GmbH (Düsseldorf, Germany). Deionized water was purified using a Milli-Q system (Millipore, Bedford, MA, USA). All other chemicals were of analytical grade. Rat liver microsomes were purchased from the Research Institute for Liver Diseases Co., Ltd. (Shanghai, China). OATP1B1-, OATP2B1-, and OATP1B3- expressing human embryonic kidney 293 (HEK293) cell lines were provided by Dr. Xiaoyan Chen from Shanghai Institute of Materia Medica, Chinese Academy of Sciences (Shanghai, China). OAT2-expressing HEK293 cell line was purchased from Shanghai Genechem Co., Ltd. (Shanghai, China). Normal human liver HL-7702 cells were purchased from the cell bank of the Chinese Academy of Sciences (Shanghai, China).

### 4.2. Preparation of Powdered ESF

Powdered ESF was prepared according to the traditional method, as previously described [8]. Briefly, the dried roots of *Sophora flavescens* were extracted twice by refluxing with 70% ethanol for 4 h. The combined extract was filtered and concentrated by rotary evaporation under vacuum at 70 °C for 3 h to obtain powdered ESF (yield of the extract: 20%). Powdered ESF (100 mg) was extracted with 50 mL of 50% methanol in an ultrasonic bath for 60 min. The extracted solution was centrifuged at 12,000 rpm for 10 min. The content of kurarinone in ESF was 171.3 ± 1.8 mg/g as determined by HPLC.

### 4.3. Animals

Male Sprague-Dawley rats (200 ± 10 g) were obtained from the Laboratory Animal Center of the Fudan University. Animal license number was SCXK (Shanghai) 2013–0016. The rats were housed in an air-conditioned room at 22 ± 2 °C and relative humidity of 50 ± 10%, under a 12 h light-dark cycle. The rats were allowed free access to food and water. The animals were acclimatized to the facilities for 7 days and fasted for 12 h with free access to water before the experiments. Animal studies were conducted according to the animal protocols and guidelines established by Ethics Committee of the Experimental Animal Care Society of Fudan University.

### 4.4. ESF Hepatotoxicity Study in Rats

The rats were divided randomly into three experimental groups (*n* = 6 per group). ESF was dissolved in 1% Tween 80 aqueous solution and administered orally (1.25 g/kg and 2.5 g/kg body weight) to the treatment groups once a day for two weeks. The vehicle control group received 1% Tween 80 solution. Blood without anticoagulants was collected for biochemistry analysis and the remaining blood samples were stored at −80 °C before analysis. After this procedure, the animals were euthanized and the liver tissues were promptly removed and weighed for calculating organ-to-body weight ratio. A portion of liver tissues was dissected and fixed in 10% formalin for hematoxylin and eosin staining, another portion was used for oil red O staining, and the remaining liver tissues were immediately frozen in liquid nitrogen until use.

### 4.5. Serum Biochemistry Analysis and Histological Analysis

Levels of triglycerides (TG), total cholesterol (CHOL), alanine aminotransferase (ALT), aspartate aminotransferase (AST), total bilirubin (TBIL), and alkaline phosphatase (ALP) in rat serum were measured with an automatic blood biochemical analyzer (Hitachi 7080, Hitachi Ltd., Tokyo, Japan). TG and CHOL in the liver were measured with triglycerides and total cholesterol assay kits, respectively (Nanjing Jiancheng Biology Engineering Institute). Liver sections were dissected, fixed in phosphate-buffered 10% formaldehyde solution and embedded in paraffin. In addition, liver sections were stained with hematoxylin and eosin and oil red O and examined for histopathological changes using light microscopy (Olympus BX53, Olympus, Tokyo, Japan).

### 4.6. Metabolomics Study

#### 4.6.1. Serum Sample Preparation

An aliquot of 50 μL serum was deproteinized with acetonitrile (200 μL) containing the internal standard (mycophenolic acid(−) 350 ng/mL, carbamazepine(+) 30 ng/mL) and the mixture was vortexed for 3 min. After centrifugation at 16,000× *g* for 10 min at 4 °C, the sample supernatants (5 μL) were injected into the UPLC-HRMS system for analysis. Quality control (QC) samples were prepared by combining equal aliquots from all serum samples in the study and were analyzed before, during, and after the run.

#### 4.6.2. Instrumentation and Conditions

The UPLC-HRMS system consisted of a Dionex UltiMate 3000 UPLC (Thermo Fisher Scientific, Germering, Germany) and a LTQ-Orbitrap Velos Pro (Thermo Fisher Scientific, Bremen, Germany). An Acquity UPLC C18 column (2.1 × 100 mm, 1.7 μm; Waters, Wexford, Ireland) was used with a mobile phase of 0.1% formic acid (A) and acetonitrile (B). The elution gradient for the serum was as follows: 10 to 40% B from 0 to 2 min, 40 to 80% B from 2 to 10 min, 80 to 90% B from 10 to 14 min, 90% B from 14 to 15 min, 90 to 10% B from 15 to 15.5 min, 10% B from 15.5 to 19.5 min. The column temperature was 35 °C, the flow rate was kept at 0.3 mL/min, and the injection volume was set at 5 μL.

HRMS experiments were performed on a LTQ-Orbitrap Velos Pro instrument equipped with an electrospray ionization source. Nitrogen was used as the sheath and auxiliary gas, and flow rates were set at 45 and 15 arbitrary units, respectively. The scan ranges were 50–1000 *m*/*z* for metabolite analysis. The spray voltages were set to 3.2 kV (−) and 3.8 kV (+) respectively, the source temperature was 300 °C, and the capillary temperature was 350 °C. Data from the LC-MS analyses were acquired using a high-resolution Fourier transform mass spectrometer with a resolution of 60,000. The highest intensity ions detected on full scan MS were selected for data-dependent scanning. MS^n^ activation parameters were isolation width 2 Da, normalized collision energy 35 V, and activation time, 10 ms. External mass calibration was applied the day before the test to ensure *m*/*z* accuracy of the mass spectrometer.

#### 4.6.3. Data Processing and Statistical Analysis

The raw data were analyzed using SIEVE software (Thermo Fisher Scientific, Cambridge, MA, USA), enabling data reduction to yield a list of mass and retention time pairs with corresponding intensities for all detected peaks. These data were exported to an Excel table and handled according to the “80% rule” and “RSD ≤ 30%” criteria [13]. The resulting data were analyzed by principal components analysis (PCA) and orthogonal partial least squares-discriminate analysis (OPLS-DA) using SIMCA-P software (version 14.0, Umetrics, Sweden). Two-dimensional scores plots were used to display the relative importance of the different sources of variation. The corresponding variable importance (VIP value) in the projection was calculated in the OPLS-DA model. A differential metabolite was selected when the VIP value was >1. Nonparametric Kruskal–Wallis rank sum test was performed to determine the significance of each metabolite using SPSS 18.0, with the significance threshold set at *p* < 0.05.

Identification of differential metabolites was based on high-resolution mass measurement (<5 ppm), MS^n^ fragment ion information, and retention time comparison with standard compounds and the following databases: HMDB (http://www.hmdb.ca), KEGG (http://www.kegg.jp), Chemspider (http://www.chemspider.com), LIPIDMAPs (http://www.lipidmaps.org), and METLIN (http://metlin.scripps.edu). Lipid compounds were identified using LipidSearch software (Thermo Fisher Scientific). Interpretation of the metabolic pathways of differential metabolites was based on metabolite correlations and the listed databases. Variation in the levels of differential metabolites between control and ESF-treated groups was visualized as heat maps.

### 4.7. Quantitative Real-Time PCR

Total RNA was extracted from liver tissues using Trizol reagent (Invitrogen, Carlsbad, CA, USA) and treated with RNase-free DNase (Invitrogen). First-strand complementary DNA (cDNA) was generated by reverse transcriptase with random primers. To evaluate the mRNA expression of carnitine palmitoyltransferase-1 (CPT-1), carnitine palmitoyltransferase-2 (CPT-2), peroxisome proliferator-activated receptor-α (PPAR-α), and long-chain acyl-CoA dehydrogenase (LCAD), real-time PCR was performed using a SYBR Green Master Mix kit and the StepOnePlus Sequence Detection System (Applied Biosystems, Foster City, CA, USA) as previously described [23]. The primers are shown in Supplementary Table S1. The 2^−ΔΔ*C*t^ method was used to determine the relative amounts of target mRNA, using β-actin as the endogenous control.

### 4.8. Effect of Kurarinone on Triglyceride Levels in HL-7702 Cells

HL-7702 cells were cultured in RPMI-1640 medium (Gibco, Grand Island, NY, USA) containing 10% fetal bovine serum (FBS) (Gibco, USA) in a humidified atmosphere of 5% CO_2_ at 37 °C. The cells were seeded in 24-well plates, cultured for 24 h, and incubated with kurarinone (1, 8, 16, 24, 32, 44, and 60 μM) dissolved in RPMI-1640 medium without FBS for 24 h. Triglyceride levels were determined using a triglyceride assay kit (Nanjing Jiancheng Bioengineering Institute) according to the manufacturer’s instructions.

### 4.9. ESF Tissue Distribution Study in Rats

Male rats were divided into five groups corresponding to five experimental time points (*n* = 3 in each group). Rats under urethane anesthesia were sacrificed by bleeding from the abdominal aorta and hepatic portal vein at 15 min, 30 min, 1 h, 2 h, and 4 h time points (3 rats/time point) after a p.o. dose of ESF (2.5 g/kg). The liver, kidneys, heart, skeletal muscles, small intestine, and bile were harvested and homogenized in threefold volume of ice-cold saline. Kurarinone levels in vivo in the plasma and liver were examined at the time points when blood biochemistry parameters were measured. Kurarinone and kurarinone glucuronides levels were detected by LC-MS/MS [24,25].

### 4.10. Uptake Transporter Study of Kurarinone and Kurarinone Glucuronides

A phenotypic study was performed using HEK293 cells that individually express OAT2, OATP1B1, OATP2B1, and OATP1B3 to identify transporters mediating the hepatic uptake of kurarinone and kurarinone glucuronides. Transporter-transfected cells were cultured in Dulbecco’s modified Eagle’s medium (Corning, Manassas, VA, USA) supplemented with 10% FBS, 2 mM l-glutamine, 1% modified Eagle’s medium nonessential amino acids solution (Gibco), 100 U/mL penicillin, and 100 μg/mL streptomycin. HEK293 cells were cultivated in a humidified atmosphere containing 5% CO_2_ at 37 °C. Time-dependent kurarinone uptake by OATP1B1, OATP2B1, OATP1B3, and OAT2 was evaluated as previously described [26]. In brief, transfected HEK293 and mock control cells were seeded on BioCoat poly-d-lysine-coated 24-well plates (BD Biosciences, San Jose, CA, USA) at a density of 2.5 × 10^5^ cells/well. After 36 h, the cells were washed twice and equilibrated in Hanks’ balanced salt solution (HBSS) for 10 min at 37 °C. The uptake was initiated by adding 0.5 mL of HBSS containing 2 μM kurarinone and terminated after 5, 10, or 30 min by aspirating the solution and washing the cells twice with 2 mL of ice-cold HBSS. The cells were lysed after three freeze/thaw cycles (−80 to 25 °C) and proteins were precipitated by methanol. LC-MS/MS [24] was used to determine the concentration of kurarinone. Total cellular protein levels were measured using a BCA protein assay kit.

Kurarinone glucuronides are the major kurarinone metabolites formed in liver microsomes of humans and rats [25]. Kurarinone glucuronides were prepared using pooled rat liver microsomes. In brief, the 100 μL reaction system consisted of rat liver microsomes (1 mg protein/mL), 50 μM kurarinone, 10 mM MgCl_2_, 5 mM d-saccharic acid 1,4-lactone monohydrate, and 25 μg/mL alamethicin in 50 mM Tris–HCl (pH 7.4). The reaction was initiated by adding UDPGA (2 mM), maintained at 37 °C for 2 h, and terminated by adding one volume of ice-cold acetonitrile. The precipitate was removed by centrifugation (16,000× *g* for 10 min) and several assays were combined for further purification [27]. Uptake transporter study of kurarinone glucuronides was carried out on OATP1B1-, OATP2B1-, OATP1B3-, and OAT2-expressing HEK293 cells as described for kurarinone. Kurarinone glucuronide levels in cells were calculated as difference in kurarinone levels after hydrolysis with β-glucuronidase [28].

## 5. Conclusions

In summary, after oral administration of ESF in rats, kurarinone glucuronides were actively transported into the liver by OATP1B3 and hydrolyzed to kurarinone, of which the concentration in hepatocytes was sufficiently high to inhibit fatty acid β-oxidation through the reduction of l-carnitine and the inhibition of PPAR-α pathway, ultimately leading to hepatic lipid accumulation and liver injury.

## Figures and Tables

**Table 1:** Identification results of serum differential metabolites in control group and *Sophora flavescens*-induced groups.

No.	t_R_ (min)	Detected *m*/*z*	Metabolites ^a^	VIP ^d^	Trend ^e^	Related Pathway ^f^	*p*-Value ^g^	ESI Mode
1	0.90	146.1647	Spermidine	1.02	↓	Glutathione metabolism	0.00	+
2	1.00	87.0084	Pyruvic acid ^b^	1.32	↓	Pyruvate metabolism	0.01	−
3	1.02	162.1115	l-Carnitine	1.96	↓	Fatty acid metabolism	0.00	+
4	1.03	118.0854	Betaine	1.66	↓	Betaine metabolism	0.03	+
5	1.04	76.0751	Trimethylamine N-oxide	1.01	↑	Choline metabolism	0.00	+
6	1.05	103.0395	3-Hydroxybutyric acid	2.13	↓	Synthesis and degradation of ketone bodies	0.00	−
7	1.06	132.0757	Creatine ^b^	2.63	↑	Glycine and serine metabolism	0.00	+
8	1.18	90.0542	Alanine	6.68	↑	Alanine metabolism	0.00	+
9	1.52	204.1219	l-Acetylcarnitine	1.20	↓	Beta oxidation of very long chain fatty acids	0.00	+
10	3.87	514.2798	Taurocholic acid ^b^	1.83	↓	Primary bile acid biosynthesis	0.01	−
11	5.39	464.2981	Glycocholic acid ^b^	2.08	↓	Primary bile acid biosynthesis	0.00	−
12	5.48	498.2852	Taurochenodesoxycholic acid ^b^	2.38	↓	Primary bile acid biosynthesis	0.00	−
13	5.68	353.2313	Prostaglandin E2 ^b^	1.13	↓	Arachidonic acid metabolism	0.00	+
14	6.73	391.2817	Ursodeoxycholic acid ^b^	3.68	↓	Secondary bile acid biosynthesis	0.00	−
15	8.41	378.2383	Sphingosine 1-phosphate	2.00	↓	Phospholipid metabolism	0.00	−
16	9.83	520.3358	LysoPC (18:2) ^c^	2.53	↑	Glycerophospholipid metabolism	0.00	+
17	10.20	544.3350	LysoPC (20:4) ^c^	2.67	↑	Glycerophospholipid metabolism	0.01	+
18	10.34	300.2883	Sphingosine	1.02	↓	Phospholipid metabolism	0.00	+
19	10.53	496.3361	LysoPC (16:0) ^c^	6.56	↑	Glycerophospholipid metabolism	0.02	+
20	11.29	522.3516	LysoPC (18:1) ^c^	2.59	↑	Glycerophospholipid metabolism	0.00	+
21	12.67	524.3668	LysoPC (18:0) ^c^	7.41	↑	Glycerophospholipid metabolism	0.00	+
22	12.78	400.3401	l-Palmitoylcarnitine	1.94	↑	Fatty acid metabolism	0.00	+

^a^ Identification of metabolites was carried out by comparison to the METLIN Metabolite Database; ^b^ Metabolites were further confirmed by comparison to reference substance; ^c^ Metabolites were further confirmed using LipidSearch software; ^d^ Selection was based on VIP values (threshold > 1); ^e^ ↑(or ↓) displays the relatively up-regulated (or down-regulated) level of the metabolite when ESF-treated group compared to control group; ^f^ Related pathway refers to METLIN, HMDB, LIPIDMAPs and KEGG Metabolite Databases; ^g^ *p*-Values were calculated by the nonparametric Kruskal–Wallis rank sum test (threshold ≤ 0.05).

**Table 2:** Tissue AUC_0–4h_ values of kurarinone and kurarinone glucuronide after oral administration of ESF (2.5 g/kg) in rats (mean ± S.D., *n* = 3).

	AUC_0–*t*_ (h·nmol/mL)							
	Protal Plasma	Body Plasma	Intestine	Liver	Kidney	Heart	Skeletal Muscle	Biliary Secretion
Kurarinone	4.4	2.4	21.4	45.5	5.4	4.7	6.9	0.8
Kurarinone glucuronide	25.0	15.3	2.2	1.4	0.7	0.1	0.4	93.3

## Supplementary Materials

The following are available online, Figure S1: Typical LC/MS total ion chromatogram (TIC) of the serum samples from (A) control, (B) ESF 1.25 mg/kg and (C) ESF 2.5 mg/kg groups under the negative ion mode (50–1000 *m*/*z*); Figure S2: Typical LC/MS total ion chromatogram (TIC) of the serum samples from (D) control, (E) ESF 1.25 mg/kg and (F) ESF 2.5 mg/kg groups under the positive ion mode (50–1000 *m*/*z*); Table S1: Sequences of primers for RT-PCR.
