# Supplementary material for: Hepatotoxicity Induced by Sophora flavescens and Hepatic Accumulation of Kurarinone, a Major Hepatotoxic Constituent of Sophora flavescens in Rats
Source: Molecules. 2017 Oct 25;22(11):1809. doi: 10.3390/molecules22111809 (PMC6150336; doi:10.3390/molecules22111809)
Supplement: Supplementary file 1 [file molecules-22-01809-s001.pdf]

Supplementary Table S1. Sequences of primers for RT-PCR

Gene		Sequences (5' to 3')
CPT-1	Forward primer	CCATAGTGCAGGAGCGTACAGT
	Reverse primer	CGAGTCCCGATGCCTTCAT
CPT-2	Forward primer	GCTCTCAAGGCTGGCATCAC
	Reverse primer	TGGAACTGAATGGAGTCAATGCT
PPAR-α	Forward primer	TACCTGTGAACACGATCTGA
	Reverse primer	GCTAGTCTTTCCTGCGAGTA
LCAD	Forward primer	GGCTGGTTAAGTGATCTCGTGAT
	Reverse primer	TCTCCACCAAAAAGAGGCTAATG
β-actin	Forward primer	GGCACCACACTTTCTACAAT
	Reverse primer	AGGTCTCAAACATGATCTGG

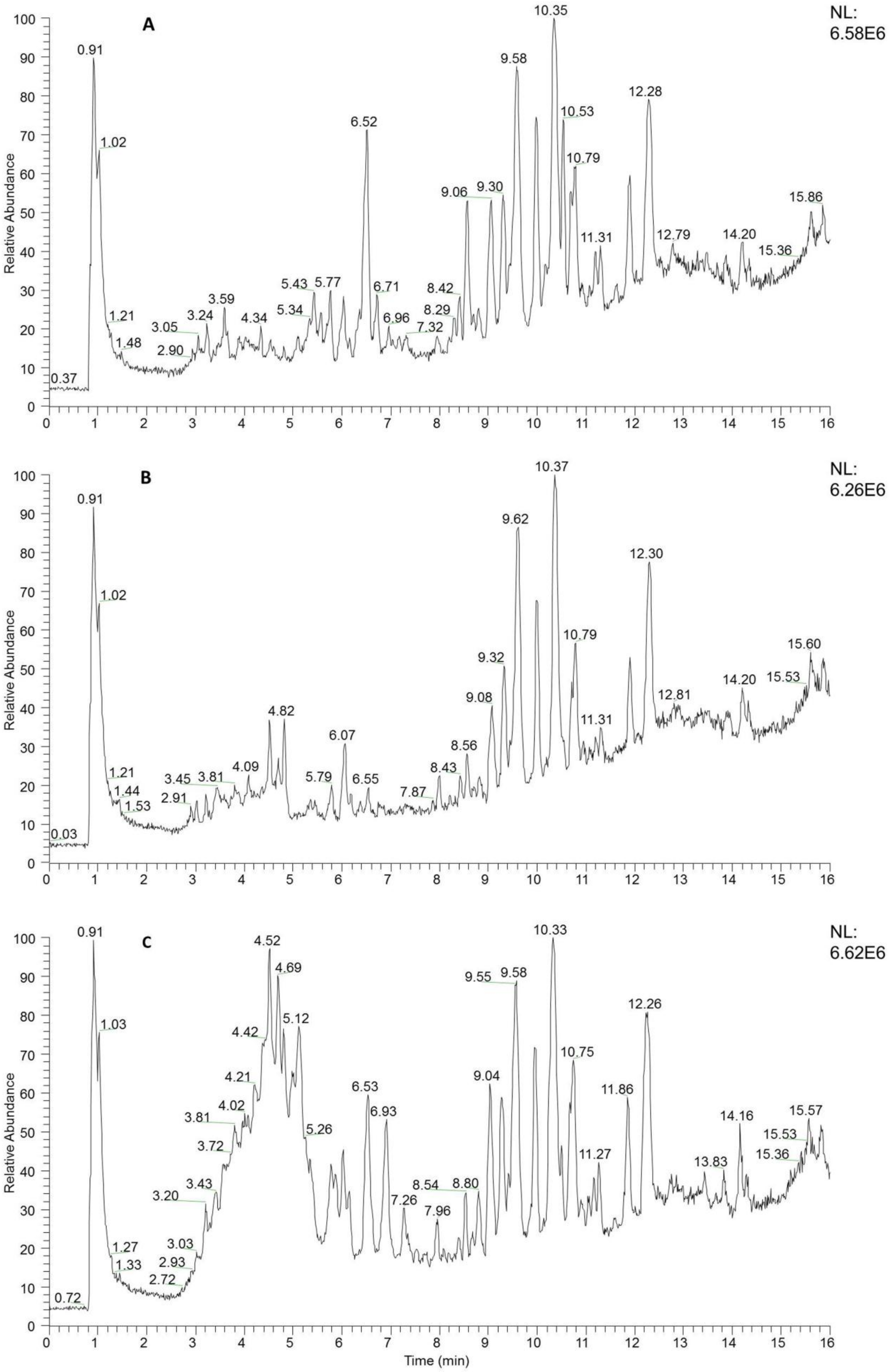

Supplementary Figure S1. Typical LC/MS total ion chromatogram (TIC) of the serum samples from (A) control, (B) ESF 1.25mg/kg and (C) ESF 2.5mg/kg groups under the negative ion mode (50–1000 m/z).

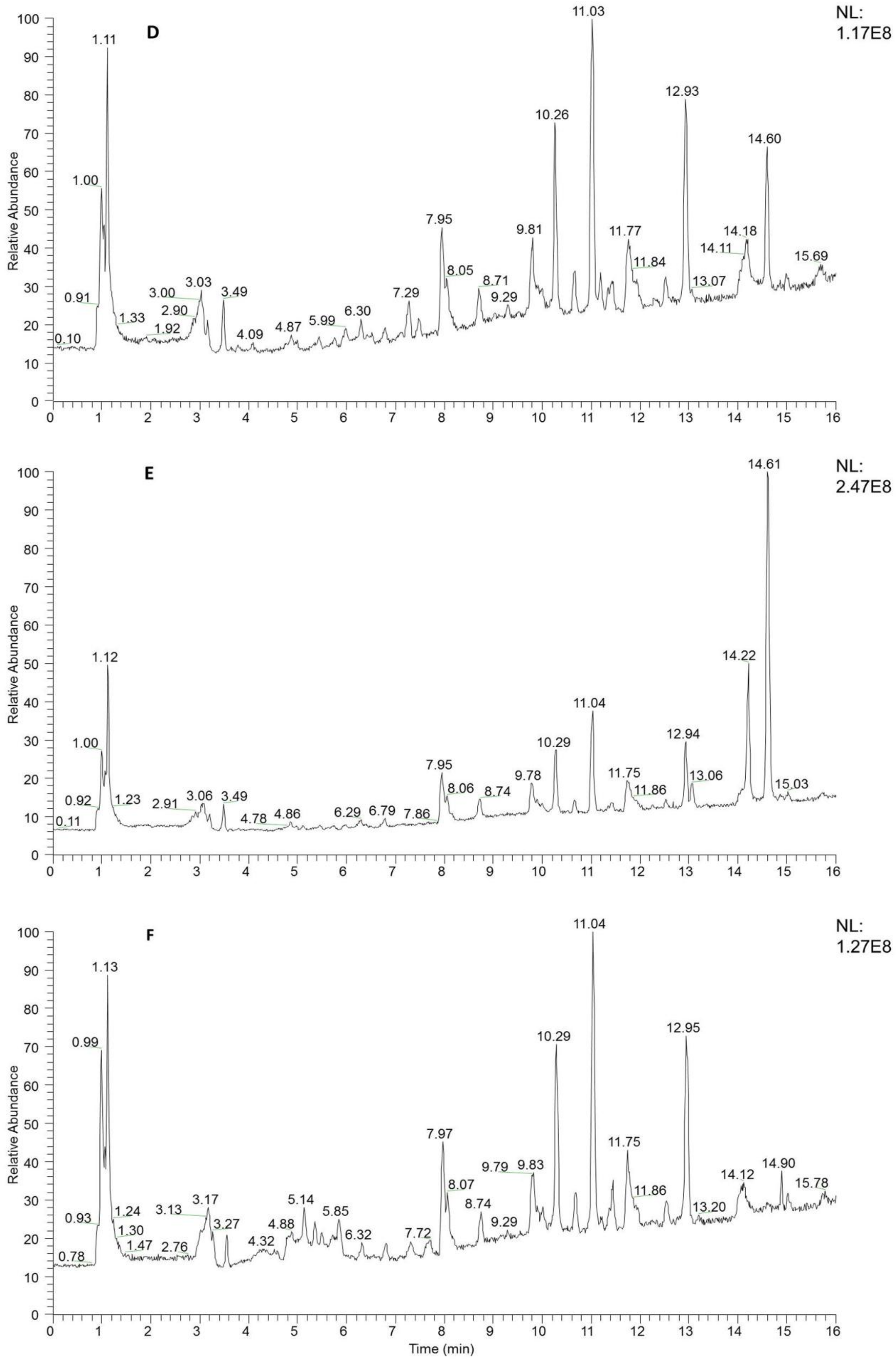

Supplementary Figure S2. Typical LC/MS total ion chromatogram (TIC) of the serum samples from (D) control, (E) ESF 1.25mg/kg and (F) ESF 2.5mg/kg groups under the positive ion mode (50–1000 m/z).
